# Editorial: Trends and perspectives for the use of crop wild relatives in crop breeding

**DOI:** 10.3389/fpls.2024.1424160

**Published:** 2024-05-21

**Authors:** Rodomiro Ortiz, Filippo M. Bassi, Mahesh Rao, Diego Rubiales

**Affiliations:** ^1^ Department of Plant Breeding, Swedish University of Agricultural Sciences, Alnarp, Sweden; ^2^ International Center for Agricultural Research in the Dry Areas, Rabat, Morocco; ^3^ Indian Council for Agricultural Research – National Institute for Plant Biotechnology, New Delhi, India; ^4^ Institute for Sustainable Agriculture, Consejo Superior de Investigaciones Científicas (CSIC), Córdoba, Spain

**Keywords:** climate change food and nutrition security, genebank, host plant resistance, resilience, resource use efficiency

Crop wild relatives (CWRs) represent a fundamental cornerstone for enhancing agrobiodiversity, fostering sustainable agriculture, and bolstering overall food and nutrition security. These invaluable genetic resources where never pampered by the help of human farmers, so still harbour a plethora of genes encoding traits crucial for adaptability to harsh environments, resilience against pathogens and pests, efficient input and resource utilization, and enhanced nutritional profiles. Their vast genetic reservoir, coupled with the diverse microbiota they host, presents a treasure for the development of more robust, nutritious, and high-yielding cultivars, thereby enriching farming systems worldwide. Despite their immense significance, a multitude of CWRs are currently imperilled, confronting threats stemming from intensive agricultural practices, rapid urbanization, environmental pollution, and the impacts of climate change. There exists a conspicuous gap in our understanding of the breadth of diversity inherent within CWRs and how this diversity can be harnessed to enhance crop improvement and agricultural practices. Regrettably, efforts toward their conservation and integration into breeding programs remain notably deficient.

In this Research Topic, there are 12 articles contributed by a total of 102 authors. Goldman et al.‘s review explores the intricate relationship between natural selection, domestication, and human-mediated evolution in determining organ shape, a crucial factor influencing consumer preference in vegetable crops. They highlight how natural selection has played a significant role in shaping organ morphology in CWRs. Through selective breeding and other genetic interventions, humans have been able to manipulate traits such as supernumerary cambia – additional layers of cambium tissue in plant stems – by exploiting allelic variation at loci controlling fundamental processes like cell division, cell elongation, transposon-mediated variation, and the partitioning of photosynthate (sugars produced during photosynthesis). Goldman et al. argue that understanding the allelic variation present in CWR is crucial for further improving and shaping organ morphology in vegetable crops. By exploring and harnessing the genetic diversity found in CWR, researchers can potentially identify novel alleles associated with desirable organ shapes. Utilizing this genetic variation through breeding programs or genetic engineering techniques could lead to the development of vegetable crops with enhanced organ shapes that better meet consumer preferences and market demands. The other review article by Verma et al. delves into the utilization of CWRs to enhance resilience in the Indian mustard [*Brassica juncea* (L.) Czern. &Coss.] cultigen pool. This paper provides a comprehensive overview of the introgression process, detailing how resistance against various stressors such as Sclerotinia stem rot, Alternaria blight, white rust, aphid infestations, drought, and high temperatures has been successfully incorporated from CWRs into *B. juncea*. Verma et al. underscore the significant challenges posed by pre- and post-fertilization barriers, particularly those arising from differences in ploidy levels, which have hindered the widespread success of germplasm enhancement endeavours. Additionally, the authors highlight the pivotal role of recent advancements in omics technologies, which may play a crucial role in improving stress resilience traits in *B. juncea*.

Kagi et al.‘s work presents a novel approach aimed at addressing the conservation challenges associated with forages on Swiss meadows. They document both the benefits and obstacles encountered while implementing their bottom-up approach. In addition to this, they also attempt to estimate the quality of conservation efforts concerning the gene pool of CWRs of forage plants. They suggest that similar strategies could be effectively employed to guide the conservation of forage CWR in other geographical regions. Furthermore, Kagi et al. discuss potential pathways to enhance CWR conservation policies, considering population dynamics and habitat levels. They likely explore strategies for integrating CWR conservation efforts into broader conservation frameworks, considering factors such as genetic diversity, population dynamics, and habitat preservation. Their holistic approach acknowledges the interconnectedness of ecosystems and the importance of preserving genetic diversity at both the species and population levels. It is worth relating the above results with those in the paper by Hagenblad et al., who shed light on the conservation dynamics of Nordic red clover (*Trifolium pratense* L.) populations, both *in situ* (in their natural habitat) and *ex situ* (in genebanks). Interestingly, the study did not find significant changes in the populations under either conservation method, indicating a degree of stability in the genetic makeup of these populations over time. Furthermore, Hagenblad et al. observed very limited gene flow from the cultivated red clover pool to the CWR populations. These results suggest that both conservation efforts can be effective in maintaining the genetic diversity of CWR populations.

The contributions by El Hanafi et al. and Keilwagen et al. highlight the potential of genomic techniques for enhancing the utilization of genebank accessions, particularly for CWRs of cereal crops such as barley (*Hordeum vulgare* L.) and wheat (*Triticum* spp. L.). El Hanafi et al. demonstrated the feasibility of genome-wide prediction using historical data on key traits such as flowering/heading date, plant height, and thousand kernel weight from barley accessions held by genebanks at the Leibniz Institute of Plant Genetics and Crop Plant Research (IPK, Gatersleben, Germany) and the International Center for Agricultural Research in the Dry Areas (ICARDA, Rabat, Morocco). By leveraging genomic data, El Hanafi et al. were able to make cross-genebank predictions, thereby improving the curation and utilization of genebank collections worldwide. This approach not only unlocks the hidden potential of genebank accessions but also addresses the information gap in genebanks, contributing to genetic enhancement programs with CWRs. Similarly, Keilwagen et al. highlight the use of genotyping-by-sequencing and whole-genome sequencing to characterize genebank accessions, focusing on wheat samples held at IPK. Through these genomic techniques, they were able to predict introgressions at a high resolution (1-Mb) across many wheat accessions. This enables researchers to identify and utilize valuable genetic variation present in genebank collections for breeding and genetic improvement efforts.

Three research articles provide insights into mapping characteristics of wheat CWRs and their potential for crop improvement. Page et al. utilized genome-wide association mapping (GWAS) to identify races in *Aegilops longissima* (primarily found in Israel) and single nucleotide polymorphisms (SNPs) associated with host plant resistance to various pathogens causing leaf, stem, and stripe rusts. The focus of the study by Debliek et al. was on mapping prehaustoria resistance against leaf rust in a biparental F_2_ population derived from crossing the wheat ancestor einkorn (*Triticum monococcum* L.) with a susceptible accession of *T. boeoticum* Boiss. They were able to find a single gene associated with resistance located at 3.4 Mbp from the peak marker within a major quantitative trait locus. In their research, Jabbour et al. located genomic regions controlling 1000-kernel weight, an important trait for drought adaptation in durum wheat. They used a segregating nested association mapping population whose recurrent parent was the Moroccan cultivar ‘Nachit’ that was derived from wild emmer (*T. turgidum* subsp. *dicoccoides* (Körn. ex. Asch. & Graebn.) Thell). These three combined articles demonstrate the utility of genomic approaches for mapping important traits in wheat CWRs, thus offering valuable insights for crop improvement efforts targeting host plant resistance, yield enhancement, and adaptation to stress-prone environments.

The research conducted by Ahmed et al. illustrates the utility of transcriptomics in dissecting host plant resistance and identifying defence-related genes in CWRs. In their study, they investigated the defence responses to *Alternaria brassicicola*, a common fungal pathogen, in both resistant white mustard (*Sinapis alba* L.) and susceptible rape (*Brassica rapa* L.). By understanding the molecular mechanisms involved in host defence responses, breeders can prioritize the selection of resistant germplasm and target specific genes or pathways for introgression into rapeseed cultivars.

The paper by Rane et al. presents findings from their investigation into the pollen structure and storage of valuable grape (*Vitis vinifera* L.). They observed significant variation in palynological traits, particularly in relation to the presence or absence of colpus, as well as in pollen dimensions. Importantly, their research also found male sterility in *V. parviflora* Roxb. and Dogridge. Furthermore, Rane et al. evaluated different temperatures for pollen storage and found that from -20°C to -196°C were effective for preserving grape pollen for up to 30 days. This finding has practical implications for the conservation and utilization of genetic resources in grape breeding programs. Moreover, this study highlights two specific CWRs, namely *V. parviflora* Roxb. × *V. vinifera* L. (Pusa Navrang) and *V. parviflora* Roxb. × *V. champini* Planc. (Salt Creek), as promising sources for rootstock breeding in grapes.

The work by Suma et al. highlights the successful utilization of interspecific hybridization and backcrossing (BS) techniques to broaden the genetic diversity of the okra (*Abelmoschus esculentus* [(L.) Moench.]) cultigen pool through the incorporation of genes from CWRs. The resulting offspring exhibited intermediate fruit morphology, indicating a mixture of traits from both the cultivated and wild parents. However, there was also evidence of some dominance of wild characteristics in the hybrids, thus suggesting successful introgression of genes from the CWR into the cultigen pool. Through further backcrossing, the researchers were able to produce offspring with fruit morphology more closely resembling the cultivated okra. This demonstrates the effectiveness of the backcrossing strategy in selecting for and fixing desired traits while eliminating undesirable wild characteristics.

The above articles within this Research Topic highlight the challenges and methodologies involved in harnessing the potential of CWRs to enhance the genetic diversity and improve the cultigen pool of cereal, fruit, oil, and vegetable crops. These papers also underscore the importance of utilizing CWRs in crop breeding programs to address current and future challenges in agriculture, such as adaptation to climate change, host plant resistance, and edible yield. Advances in breeding methods and genomics offer unprecedented options to unlock the genetic potential of CWRs and incorporate their valuable traits into crops, thereby contributing to the sustainable improvement of global food and nutrition security as well as agricultural productivity.

## Author contributions

RO: Conceptualization, Writing – original draft, Writing – review & editing. FB: Conceptualization, Writing – original draft, Writing – review & editing. MR: Conceptualization, Project administration, Writing – original draft, Writing – review & editing. DR: Conceptualization, Writing – original draft, Writing – review & editing.

